# Patient characteristics of medical encounters at the Olympic Stadium during the Tokyo 2020 Olympic and Paralympic games

**DOI:** 10.3389/fpubh.2025.1674017

**Published:** 2025-10-27

**Authors:** Yumiko Sekizaki, Akina Hiden, Ryo Yamamoto, Yusho Nishida, Akihiko Kondo, Hiroki Takami, Yoichi Toyomoto, Hiroyuki Ishida, Shin Watanabe, Junichi Sasaki

**Affiliations:** ^1^Department of Nursing, Keio University Hospital, Tokyo, Japan; ^2^Department of Emergency and Critical Care Medicine, Keio University School of Medicine, Tokyo, Japan; ^3^Emergency and Intensive Care Unit, Juntendo University Nerima Hospital, Tokyo, Japan; ^4^Health Screening, Rehabilitation and Fitness Unit, Medical Support Division, Juntendo University Hospital, Tokyo, Japan; ^5^Sports Medical Research Center, Keio University, Yokohama, Japan; ^6^Department of Emergency and Disaster Medicine, Juntendo University School of Medicine, Tokyo, Japan

**Keywords:** Olympics, post Olympic reviews, mass gathering, first aid, Tokyo, hospitals, delivery of health care

## Abstract

**Objective:**

Mass gatherings challenge local healthcare systems due to increased spectator demands. During the Tokyo 2020 Olympic and Paralympic Games, a medical team stationed at the Olympic Stadium implemented an on-site medical system for over 3,000 nonathlete attendees. This study analyzed medical encounters among nonathlete participants and evaluated the system’s effectiveness.

**Methods:**

A retrospective descriptive analysis was conducted, reviewing medical records from the on-site medical suite at the Tokyo 2020’s main stadium and from hospitals to which patients were transferred. Data included patient demographics, symptoms, vital signs, diagnoses, treatments, and response times both on-site and at hospitals.

**Results:**

Of the 44 patients included, heat-related illness and trauma were the most frequent conditions. Seven (15.9%) patients required hospital transport. Median times recorded were 22 min to the on-site suite, 51 min under the on-site care, and 66 min of prehospital time.

**Conclusion:**

The availability of specialized physicians and nurses facilitated efficient triage and early treatment within the on-site setting. This study underscores the importance of medical preparedness for future large-scale gatherings.

## Introduction

Mass gathering events, like the Olympic and Paralympic Games, can strain local healthcare systems due to the influx of spectators (1–5). On-site medical systems have been proposed as an effective solution for managing the temporary population surge, though no standardized system exists to ensure adequate on-site medical care. Previous studies examining mass gatherings have focused on casualty rates (6, 7), hospital transfer rates (8), and diagnoses (9, 10), but most sporting event analyses primarily address athletes, not spectators (1–5, 11, 12).

Before the Tokyo 2020 Olympic and Paralympic Games, the medical team at the Olympic Stadium (OLS) developed an on-site medical system based on prior event reports, employing Healthcare Failure Mode and Effect Analysis (HFMEA) (13). Although the games were held without general spectators, essential nonathlete participants remained, making medical care necessary. Consequently, the team adapted the on-site medical system, simplifying it by eliminating the first responder system for spectators and reducing the number of medical staff, to effectively serve this unique setting during Tokyo 2020.

It is essential to review the outcomes of patients who required medical care at the OLS to build a foundation for on-site medical systems at large-scale sports events like the Olympic and Paralympic Games. This paper presents a comprehensive data on medical encounters with nonspectator, nonathlete participants at the OLS throughout the Tokyo 2020 period. To develop a foundational on-site medical system for sports events, we analyzed detailed medical records, including the number and types of cases, on-site treatments administered, and clinical outcomes following hospital transfers.

## Methods

### Study design and settings

A retrospective descriptive study was conducted using medical records from the on-site medical suite at Tokyo 2020’s OLS for nonathlete participants. This clinic was established by the Tokyo Organizing Committee of the Olympic and Paralympic Games (TOCOG) in compliance with International Olympic Committee guidelines before the start of Tokyo 2020.

TOCOG prepared on-site medical care across all 41 Tokyo 2020 venues. The OLS, serving as the main stadium for most athletic events and the opening and closing ceremonies, has a capacity of 680,000 spectators. Medical personnel, including the Venue Medical Officer (VMO), Deputy Venue Medical Officer, and other healthcare providers, were gathered from nearby medical facilities, including two university hospitals. The on-site medical system was created by the medical team using HFMEA to meet the needs of nonathlete, nonspectator participants without overburdening local healthcare resources.

Due to the COVID-19 pandemic, Tokyo 2020 was postponed for 1 year. TOCOG decided to hold the Olympic Games from July 23 to August 8, 2021, without spectators. The Paralympic Games followed from August 24 to September 5, 2021, with limited attendance of several 100 students. Despite the absence of general spectators, over 3,000 nonathlete, nonspectator participants worked at the OLS daily during the events. Approval for human research was obtained from Institutional Review Board of Keio University School of Medicine (application no: 20211049). Informed consent was waived because of the anonymity of the data.

### On-site medical system at the OLS

The system established at the OLS included a main medical suite, termed the Key Station (KS), satellite locations where medical personnel were on standby, and designated rooms for VIPs. These medical suites operated a few hours before and after each athletic event or ceremony.

When a patient was identified, the KS was alerted by phone. A doctor or nurse was then dispatched from either the KS or a satellite area to triage the patient. Using a structured ABCDE approach based on symptoms and vital signs, the dispatched personnel assigned a code to the patient and reported it to the KS (Table 1). For the patients coded RED, a mobile medical unit (MMU), consisting of a doctor and nurse with resuscitation equipment and medications, was sent from the KS, while the nearest tertiary hospital was informed, and an ambulance was activated. For YELLOW-coded patients, the MMU was dispatched, but no ambulance was activated. GREEN-coded patients were escorted to the KS by the triaging personnel. All patients were treated at the KS until discharge or hospital transfer.

**Table 1:** Coding criteria.

Code	Criterion
RED	Cardiopulmonary arrest
	Airway obstruction
	Severe dyspnea
	Suspected cardiac chest pain
	Glasgow coma scale ≦ 8
	Suspected stroke
	Seizure
	Anaphylaxis
	High-energy mechanism of injury
YELLOW	Neither RED nor GREEN code
GREEN	ABCDE assessment clear* and patient is ambulatory

*No critical symptoms related to airway, breathing, circulation, disability (central nervous system impairment), or body surface.

The KS was equipped with electrocardiography and COVID-19 polymerase chain reaction (PCR) testing, but blood tests, urinalysis, and imaging were not available. Intravenous (IV) fluids and various medications were available at the KS and VIP rooms, though not at the satellite areas.

### Study population

Patients at the OLS during the entire Tokyo 2020 period were included, regardless of their treatment needs. However, we excluded those who were not assessed by any healthcare providers within the on-site medical system.

### Data collection and definition

Data were gathered from medical records at the KS of the OLS. The available patient information included age, sex, chief complaint, vital signs, codes assigned at the scene, transfer methods to the KS, diagnostic tests and treatments administered at the KS, final diagnoses, disposition from the KS, methods of hospital transfer, and a timeline of all medical interactions.

The scene time interval was defined as the period between the notification call to the KS and the patient’s arrival at the KS. Total on-site time was the duration from the call to the KS until discharge. Ambulance dispatch time was the interval from the call to the KS until the ambulance’s arrival. Total prehospital time was defined as the duration from the call to the KS until the patient arrived at the hospital.

### Outcome measures

The outcomes of this descriptive study included (1) the number and specialties of medical personnel within the on-site medical system at the OLS and (2) patient data such as symptoms, presentation time, assigned codes, diagnoses, medical procedures performed, patient disposition, scene time interval, length of stay at the KS, total on-site time, ambulance dispatch time, total prehospital time, length of stay in the emergency department, and the number of patients requiring transfer.

### Statistical analysis

Descriptive statistics are presented as median (interquartile range) or number (percentage). All statistical analyses were performed using SPSS, version 27.0 (IBM, Armonk, NY, USA), and Microsoft Excel (Microsoft, Redmond, WA, USA).

### Patient and public involvement

No patients or members of the public were involved in the study design, recruitment, conduct, or interpretation of the results.

## Results

During the study period, 46 patients were identified at the OLS, but 2 were not assessed by medical personnel due to closure of the medical suites. Consequently, 44 patients were included in the study.

Throughout Tokyo 2020, the on-site medical system at the OLS comprised 38 doctors and 42 nurses (Supplementary Table S1). Most of the participating doctors specialized in Emergency Medicine, Internal Medicine, or Surgery, with 12 (31.6%), 11 (28.9%), and 7 (18.4%), respectively. The allocation of doctors and nurses in each designated medical area is detailed in Table 2. The VIP medical rooms were only operational during ceremony days and major events, resulting in a higher number of medical personnel on those days compared to regular days.

**Table 2:** Medical personnel distribution by medical suite.

Event type	Number of medical personnel					
	KS		VIP		Satellite	
	MD	RN	MD	RN	MD	RN
Ceremony rehearsal	3	1	0	0	0	0
Ceremony	4	1	3	1	1	1
Key event*	3	1	1	1	2	0
Normal event	4	1	0	0	1	1
Normal event with spectators	3	0	0	0	1	1

KS, key station; VIP, very important person lounge medical station; MD, medical doctor; RN, registered nurse.

*Men’s and women’s 100-m finals and men’s 200-m final.

Table 3 outlines the patient characteristics at the OLS. The median age of the patients was 24 (20–49), and the majority were female [33 (75.0%)]. Most patients had no comorbidities. Of the 32 patients coded on-site, 18 (56.3%) were coded by a doctor. Only one patient (3.1%) received a RED code, while over half were coded YELLOW. Seven (15.9%) patients required hospital transfer (Table 3).

**Table 3:** Patient characteristics at the Olympic Stadium.

Patient details	n	rate
Total cases, *n*	44	
Demographics		
Age (years), median (IQR)	24	(20–48.5)
Age < 18 years, *n* (%)	5	(11.4%)
Sex (male), *n* (%)	11	(25%)
Past medical history, *n* (%)		
Hypertension	2	(4.6%)
Diabetes mellitus	0	(0.0%)
Heart disease	2	(4.6%)
Psychiatric disease	5	(11.4%)
Code, *n* (%)		
GREEN	12	(37.5%)
YELLOW	19	(59.4%)
RED	1	(3.1%)
Coded on scene, *n* (%)	32	(72.7%)
Coding medical staff, *n* (%)		
Physician	18	(56.3%)
Nurse	14	(43.7%)
FT dispatch, *n* (%)	19	(59.4%)
Simultaneous dispatch of coding staff, *n* (%)	4	(9.09%)
Timing of visit, *n* (%)		
Morning (6 am–9 am)	1	(2.3%)
Daytime (9 am–6 pm)	21	(47.75)
Night (6 pm–1 am)	22	(50.0%)
Hospital transfer, *n* (%)	7	(15.9%)

KS, key station; FT, field team; IQR, interquartile range.

Table 4 presents the medical procedures, diagnoses, and timelines of all medical interactions. The most frequent diagnosis was heat illness, affecting 16 (36.4%) patients, while trauma was identified in 10 (22.7%) patients. Five patients exhibited COVID-19-related symptoms, with four (9.1%) having a fever and one (2.3%) presenting respiratory symptoms without fever. The median times for arrival at the KS, duration of on-site medical care, and total prehospital time were 22 (17–28), 51 (46–82), and 66 (54–77) min, respectively.

**Table 4:** Diagnosis and duration of care.

Diagnosis	*n*	rate
Endogenous etiology	16	(36.3%)
Arrhythmia	1	(2.3%)
Syncope/presyncope	4	(9.1%)
Infectious disease	3	(6.8%)
Hyperventilation	2	(4.5%)
Other	6	(13.6%)
Exogenous etiology	28	(63.6%)
Heat illness	16	(36.4%)
Trauma	10	(22.7%)
Other	2	(4.5%)
COVID-19-related symptoms, *n* (%)		
Fever	4	(9.1%)
Respiratory symptoms	1	(2.3%)
Treatment/test, *n* (%)		
IV fluid	13	(29.5%)
IV medication	3	(6.8%)
PO fluid	14	(31.8%)
PO medication	2	(4.5%)
SARS-CoV-2 PCR test	2	(4.5)
Duration of care	Min	Median (IQR)
Time to KS arrival, min, median (IQR)	22	(17–28)
Length of KS stay, min, median (IQR)	36	(25–67)
Total duration under on-site medical care, min, median (IQR)	51	(46–82)
Time to ambulance arrival at KS, min, median (IQR)	42	(29–45)
Time to hospital arrival, min, median (IQR)	66	(54–77)
Length of ER stay, min, median (IQR)	68	(60–138)

IV, intravenous; PO, per oral; SARS-CoV-2, severe acute respiratory syndrome coronavirus 2; PCR, polymerase chain reaction; KS, key station; IQR, interquartile range; ER, emergency room.

## Discussion

In this study, all patients requiring medical care were effectively treated with minimal prehospital time, indicating the successful implementation of an on-site medical system that utilized triage codes and the ABCDE approach. Notably, although the hospital transfer rate for patients was higher than in previous studies of other on-site medical systems (6–9, 13–16), the time spent at the site was shorter than reported elsewhere.

The short duration of stay at the KS (only 36 min) likely resulted from the well-established triage systems implemented prior to Tokyo 2020. Additionally, the emergency room (ER) stay for patients transferred from our KS was approximately 1 h, significantly less than the average ER stay within the Japanese medical system (17). This suggests that the current on-site medical system not only effectively identified patients needing hospital transfer in advance but also allowed on-site healthcare providers, including registered nurses and specialized physicians, to manage a wide range of medical conditions at the KS. Furthermore, patients triaged as RED received immediate treatment from emergency physicians on-site, including medication, which is typically not feasible under the current Japanese emergency medical system management.

Importantly, the current on-site medical system was developed with formal vulnerability analyses that anticipated several hazards during mass-gathering events and actual patient encounters (13). This analysis was known as HFMEA that has been used for the quality improvement of health care systems and were validated in several studies. Interestingly, as we reported previously, the onsite medical system for mass-gathering events could have significant vulnerabilities (potential failures) on misidentification of patient by first responders, delayed immediate care by the MMU at the scene, misjudgment of disposition from the on-site medical suite, and inappropriate care during transportation to hospital, rather than event-specific or constantly changing variables over time (13). Based on these vulnerabilities, the current system in TOKYO2020 consisted of health care providers who were well trained to mitigate the potential failures particularly by obtaining skills on immediate care at the scene and during transportation. However, first responders were omitted from the current system because of no spectators in the venue, and therefore the corrective actions for appropriate identification of patients could not be evaluated in this study.

Unfortunately, the Olympic and Paralympic Games Tokyo 2020 were held without spectators, apart from several 100 students, despite our system being designed to accommodate over 75,000 individuals, including spectators and nonathlete staff. As a result, our findings could not fully validate the safety and efficacy of the current on-site medical system. However, we adapted the system by reducing the number of healthcare providers to one-tenth of the original plan, indicating that the system could be implemented at a large-scale event. In addition, we conducted a vulnerability analysis of this on-site medical response system prior to its use in Tokyo 2020, which indicated that the quality of the on-site medical system relies heavily on nonprofessional individuals present at the venue to identify sick patients. Although the optimal number of healthcare providers is difficult to be determined, this on-site system can be one of practical models for mass-gathering events and should be further examined.

Another factor affecting the generalizability of this system is the regional epidemiology of diseases. In the event of a pandemic, such as COVID-19, the on-site medical system would need modifications for infection control. This could involve isolating patients with infectious symptoms and providing designated medical kits (e.g., PCR tests) to healthcare providers on-site, which may alter the triage protocol (18–20). In addition, climate conditions can influence spectators’ physical health and baseline risks for specific diseases, such as heat-related illnesses (6, 21, 22). Therefore, preparing for region-specific diseases is a crucial adjustment for the proposed one-site medical system: Examples of the adjustment of current system would include increasing the number of health care providers under the infectious disease pandemic (providers easily take sick leave) and preparing the continuous care during long transportation to hospital (prehospital transportation system is likely compromised). As the vulnerabilities on the system depend on the high severity and probability of failures or single-point weakness, event/hazard specific modifications can be discussed using the on-site medical system presented in this study.

### Limitations

This study has several limitations. First, the on-site medical system was organized by doctors and registered nurses from three designated institutions during Tokyo 2020. The established communication among healthcare providers with known backgrounds could potentially overstate the efficacy of the current system. Second, all members of the on-site medical team received training in triage coding and participated in simulations at the venue before the event, which may have improved coding accuracy and expedited patient transport to the KS. Third, since Tokyo 2020 received support from the Tokyo city government, transportation from the venue was well coordinated. Finally, as the current on-site system was evaluated only with the event without any spectators, this study could not analyze the impact on local emergency medical services nor surrounding hospitals. Therefore, further studies should be conducted to validate this system at other mass gathering events that are fully occupied with spectators.

## Conclusion

The on-site medical system at the OLS during Tokyo 2020 successfully handled medical encounters. The predominant diagnoses were heat illness and trauma, and the availability of specialized medical personnel facilitated prompt triage and treatment, leading to significantly shorter ER waiting times. Further validation of this on-site medical system is necessary for other mass gathering events.

## Data Availability

The original contributions presented in the study are included in the article/Supplementary material, further inquiries can be directed to the corresponding author.
