# Supplementary material for: Patient characteristics of medical encounters at the Olympic Stadium during the Tokyo 2020 Olympic and Paralympic games
Source: Front Public Health. 2025 Oct 27;13:1674017. doi: 10.3389/fpubh.2025.1674017 (PMC12597977; doi:10.3389/fpubh.2025.1674017)
Supplement: Supplementary file 1 [file Table_1.docx]

| **Supplemental Table S1. Qualifications and specialties of medical staff** | | | |
| --- | --- | --- | --- |
| **Doctor, n (%)** | Total | 38 |  |
|  | Emergency Medicine | 12 | 31.6% |
|  | Internal Medicine | 11 | 28.9% |
|  | General Surgery | 7 | 18.4% |
|  | Obstetrics/Gynecology | 1 | 2.6% |
|  | Orthopedic Surgery | 3 | 7.9% |
|  | Pediatrics | 2 | 5.3% |
|  | Plastic Surgery | 1 | 2.6% |
|  | Psychiatry | 1 | 2.6% |
| **Nurse, n** | Total | 42 |  |
